# Review of Cases of E-Cigarette or Vaping Product Use-Associated Lung Injury (EVALI) and Brief Review of the Literature

**DOI:** 10.1155/2020/1090629

**Published:** 2020-06-14

**Authors:** Amr Essa, Jeffrey Macaraeg, Nikhil Jagan, Daniel Kwon, Saboor Randhawa, Matthew Kruse, Stanley Thomas, Manasa Velagapudi, John Horne, Shraddha Narechania, Michael Kaster, Carrie Valenta, Venketraman Sahasranaman, Douglas Moore

**Affiliations:** ^1^Creighton University School of Medicine, Department of Internal Medicine, Omaha, USA; ^2^Creighton University School of Medicine, Department of Pulmonary, Critical Care, and Sleep Medicine, Omaha, USA; ^3^Creighton University School of Medicine, Department of Pathology, Omaha, USA; ^4^Creighton University School of Medicine, Department of Radiology, Omaha, USA; ^5^Creighton University School of Medicine, Department of Infectious Diseases, Omaha, USA

## Abstract

Since the appearance of the E-Cigarette in the early 2000s, its industry, popularity, and prevalence have risen dramatically. In the past, E-Cigarette use with the vaping of nicotine or cannabis products had been associated with a few reported cases of lung injury. However, in 2019, thousands of cases of E-Cigarette or vaping product use-associated lung injury (EVALI) were reported in the United States. Evidence linked this outbreak with vaping of tetrahydrocannabinol (THC). We report two confirmed cases of EVALI and their associated clinical, radiologic, and pathologic features. This report supports the growing body of information regarding EVALI. It also discusses various substances, particularly vitamin E acetate, which has been suggested as a causative agent.

## 1. Introduction

The E-Cigarette is a battery-operated device used to produce an aerosol that is inhaled into the lungs, the composition of which is determined by the content of the E-Liquid. It usually delivers nicotine; however, it can be used to deliver other substances such as tetrahydrocannabinol (THC), cannabidiol (CBD), and butane hash oils [1]. Since the introduction of E-Cigarette into the US market in 2007, there have been a few published and documented cases which describe lung disease in relation to vaping of nicotine or extracts of cannabis, with the earliest documented in 2012 [2–5]. The summer of 2019 witnessed an alarming upsurge in the number of vaping-induced lung injury cases. The increasing mortality and associated morbidity catapulted it to national attention and led to the coining of the term E-Cigarette or vaping product use-associated lung injury (EVALI) [6]. Despite the prevalence of vaping in the US and other developed countries, such as the United Kingdom, wherein 3.2 million people vape, an outbreak of this magnitude had never occurred previously [7]. Collaboration between the Centers for Disease Control and Prevention (CDC), state health departments, the medical communities, and the public is ongoing to further delineate the nature of this outbreak and the causative agents. An association between EVALI and non-nicotine-containing liquids has been suggested. Although it remains unclear which group of chemicals are involved, vitamin E acetate recently has been implicated. In this report, we highlight two cases of EVALI which were diagnosed and treated at our institution. We further discuss the clinical, radiologic, and pathologic features, with a brief review of the current body of published literature.

## 2. Case Presentation

### 2.1. Case 1

**Figure 1:**
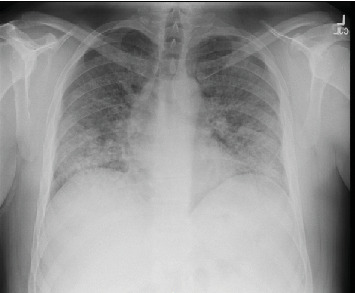
Case 1 CXR on presentation.

**Figure 2:**
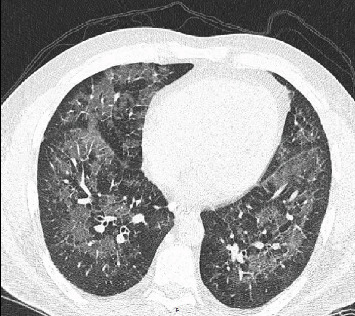
Case 1 chest CT scan on presentation (axial view).

**Figure 3:**
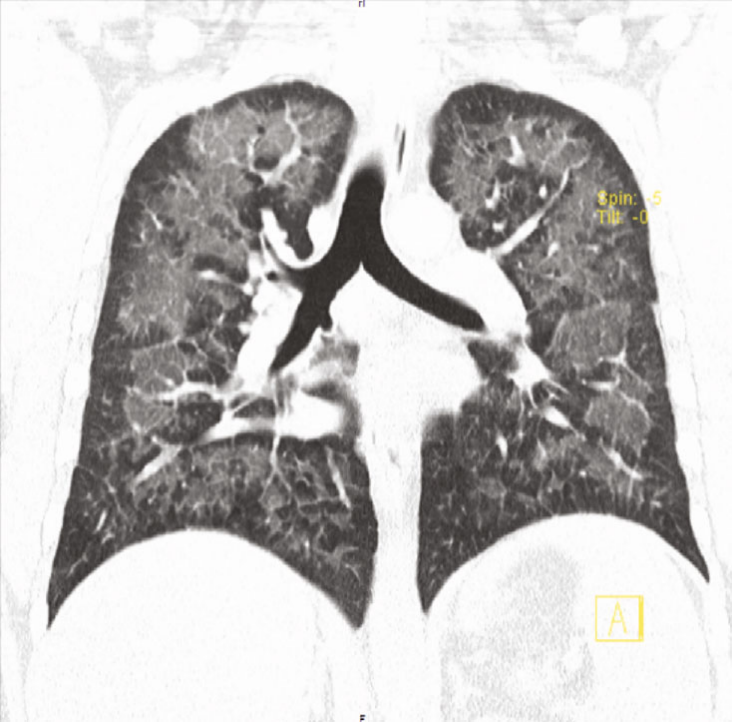
Case 1 chest CT scan on presentation (coronal view).

**Figure 4:**
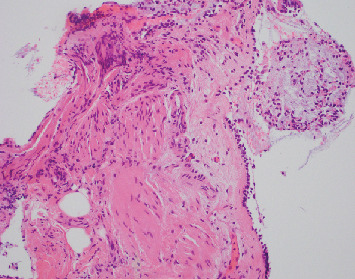
Case 1 transbronchial biopsy of upper division of left upper lobe with benign bronchial mucosa.

**Figure 5:**
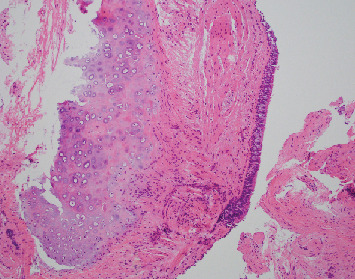
Case 1 transbronchial biopsy of left lingula with benign bronchial mucosa.

A 42-year-old man with no underlying medical problems presented with a two-week history of worsening nonproductive cough, subjective fever, chills, and diaphoresis. The history was significant for vaping THC once or twice weekly for the last one year. He reported no use of tobacco, alcohol, or illicit drugs. He denied any recent travel, and he had a desk job with no reported occupational exposures. On presentation, he was hypoxic with an oxygen saturation of 85% on room air that improved with one liter of oxygen via nasal cannula. He was afebrile and hemodynamically stable. Physical exam was significant for diffuse bilateral rales. Chest radiograph (CXR) revealed diffuse interstitial prominence (Figure 1). A subsequent computed tomography (CT) scan of the chest revealed extensive symmetric ground-glass opacities in all lobes with interlobular septal thickening and subpleural sparing (Figures 2 and 3). He was started on broad-spectrum antimicrobial therapy with azithromycin, ceftriaxone, and levofloxacin to treat potentially bacterial pneumonia, all of which was discontinued after cultures were sterile (Table 1). Bronchoalveolar lavage (BAL) of the right middle lobe was performed. A transbronchial tissue biopsy of the left upper lobe (upper division and lingula) (Figures 4 and 5) revealed benign bronchial mucosa without significant pathologic abnormality. Since an infectious etiology was ruled out, he was discharged on prednisone 40 mg daily with tapering over four weeks. He reported improvement of symptoms within one week after discharge.

### 2.2. Case 2

**Figure 6:**
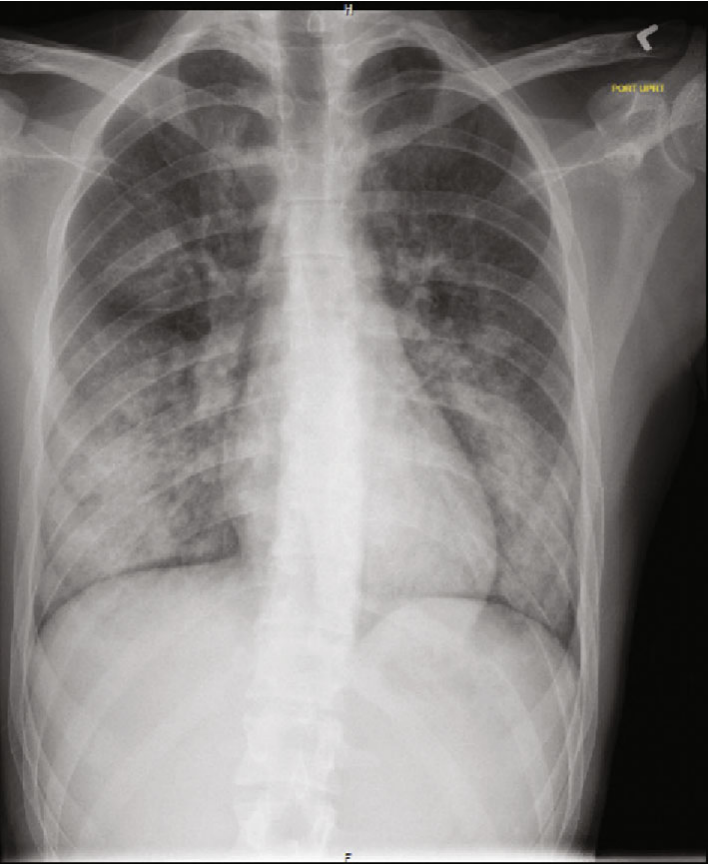
Case 2 CXR on presentation.

**Figure 7:**
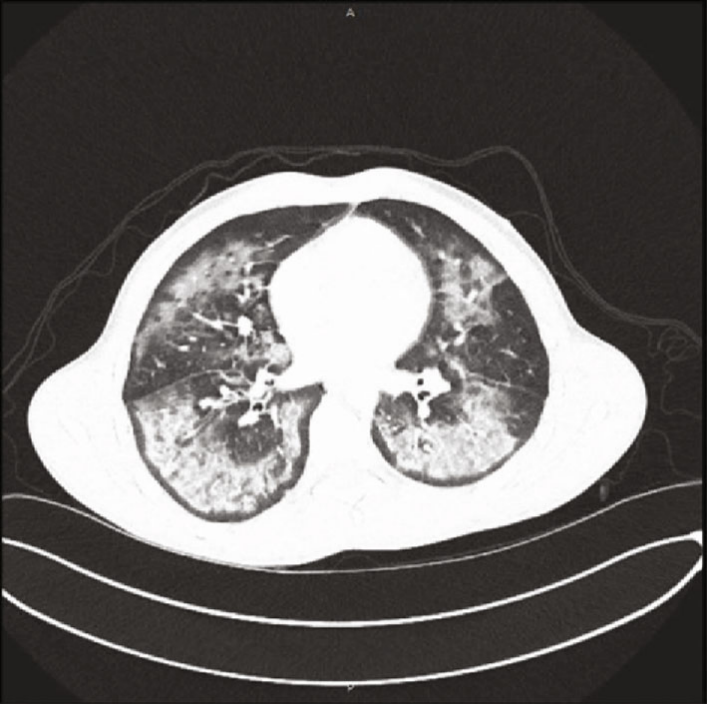
Case 2 chest CT scan on presentation (axial view).

**Figure 8:**
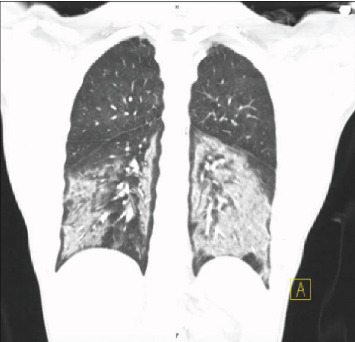
Case 2 chest CT scan on presentation (coronal view).

**Figure 9:**
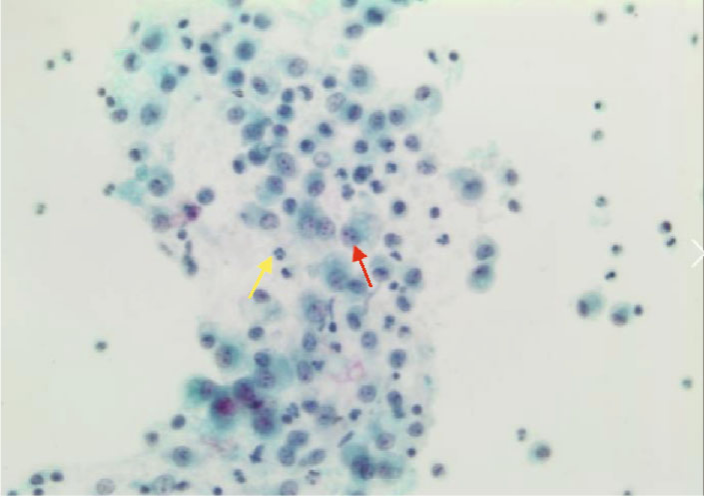
Case 2 bronchioalveolar lavage (BAL) of right lower lobe. Macrophages (red arrow) and neutrophils (yellow arrows).

**Figure 10:**
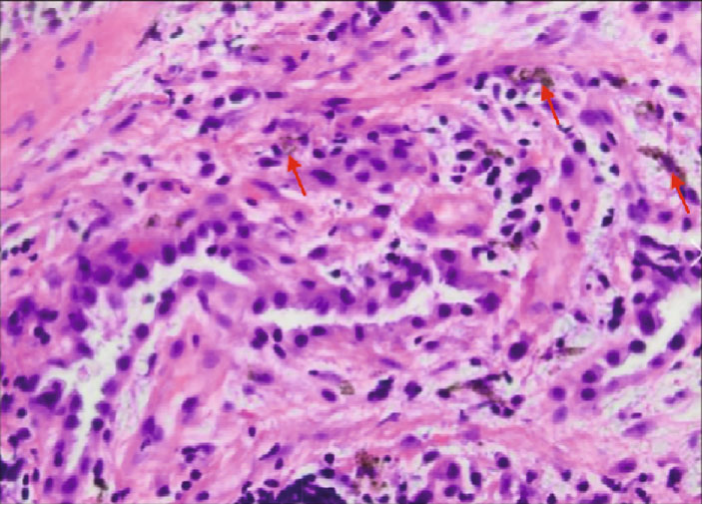
Case 2 transbronchial biopsy of right lower lobe. Macrophages containing blackish brown pigmented particles (red arrows).

**Figure 11:**
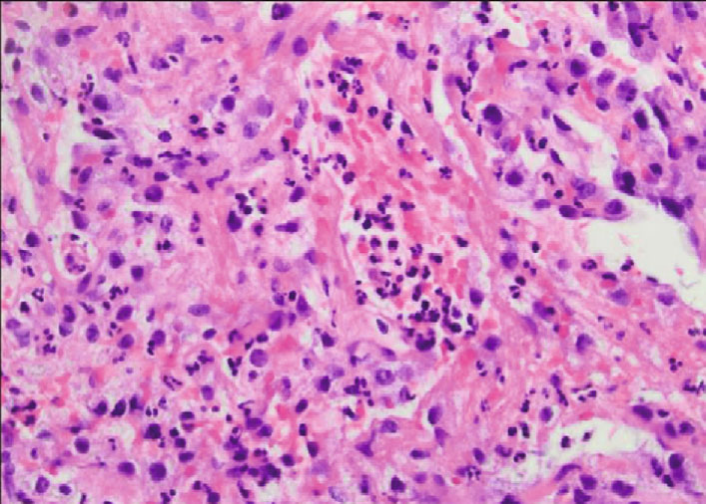
Case 2 transbronchial biopsy of right lower lobe with multiple neutrophils.

**Figure 12:**
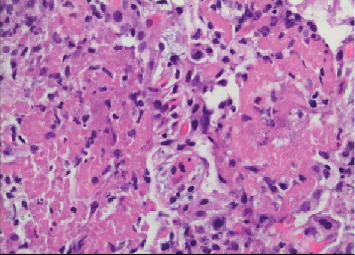
Case 2 transbronchial biopsy of right lower lobe. Eosinophilic fibrin exudate between cells, typical for pneumonia.

**Figure 13:**
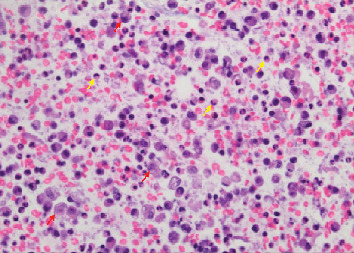
Case 2 transbronchial biopsy. Macrophages (red arrows) and neutrophils (yellow arrows) in a background of exudative material.

**Figure 14:**
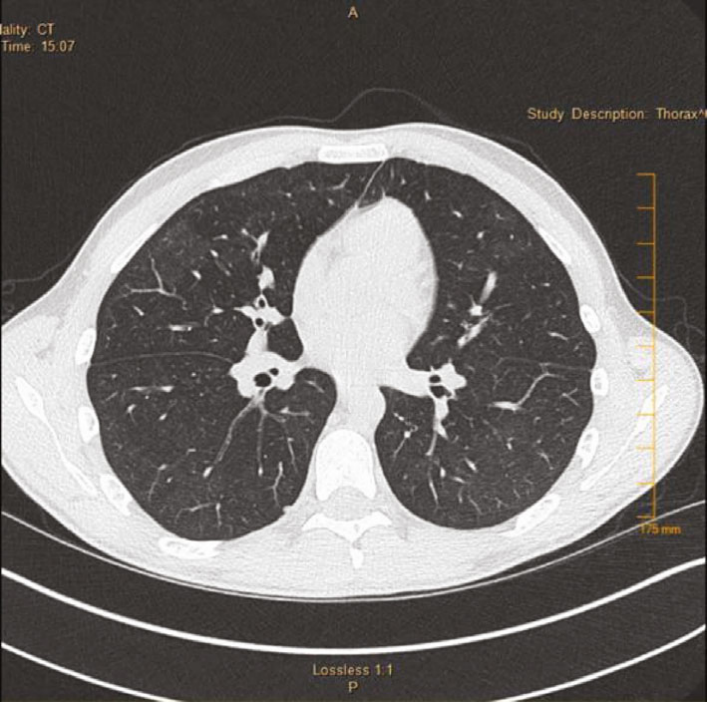
Case 2 chest CT scan 6 weeks post discharge (axial view).

**Figure 15:**
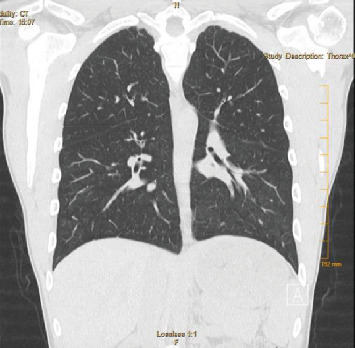
Case 2 chest CT scan 6 weeks post discharge (coronal view).

A 21-year-old man with a history of exercise-induced asthma presented with a one-week history of worsening productive cough, dyspnea, nausea, vomiting, and malaise. He stated that he vaped THC once every hour while awake for the last three months and once daily for the previous year. He reported no use of tobacco products, alcohol, or other illicit drugs. He reported recent travel to the Southwestern United States and Minnesota the month prior to hospitalization. He denied any occupational exposure. On presentation, he was hypoxic with an oxygen saturation of 88%, which improved with two liters of oxygen via nasal cannula. He was afebrile and hemodynamically stable. Physical exam was significant for bibasilar crackles. CXR on admission showed patchy alveolar opacities in the mid and lower lungs bilaterally (Figure 6). CT chest demonstrated basal-predominant bilateral ground-glass opacities with subpleural sparing, in addition to pneumomediastinum and slightly prominent bilateral hilar lymph nodes (Figures 7 and 8). He was started on broad-spectrum antimicrobial therapy with vancomycin, piperacillin-tazobactam, azithromycin, and fluconazole with coverage to treat potential coccidioidomycosis and presumed bacterial pneumonia, all of which were discontinued after cultures were sterile (Table 1). BAL of the right lower lobe revealed macrophages with neutrophils and lymphocytes (Figure 9). A transbronchial tissue biopsy of the right lower lobe showed an acute fibrinous and organizing pneumonia pattern with no prominent eosinophils, granulomas, or malignancy (Figures 10–13). Six-minute walk before discharge was notable for oxygen desaturation with activity, and subsequently, he was discharged on one-liter oxygen via nasal cannula and prednisone 40 mg daily tapering over four weeks. He reported a resolution of his symptoms at the time of his clinic follow-up visit about six weeks following discharge. Repeat chest high-resolution CT (HRCT) at that time showed remarkable improvement in the aeration of his lungs and resolution of the bilateral ground-glass opacities (Figures 14 and 15).

## 3. Discussion

Since the 2000s, when E-Cigarette made its initial appearance, the vaping industry has shown rapid emergence. Its popularity and prevalence have been driven by an apparent improved nicotine delivery system with attractive features for users and a wide variety of flavors. E-Cigarette marketing-related expenditure in the United States increased from $3.6 million in 2010 to $125 million in 2014 [7]. At the same time, the prevalence of vaping has been on the rise, with more than a quarter (25.4%) of 12th-grade students in 2019 saying that they had vaped in the last 30 days, more than double the rate in 2017 [8]. A study developed to describe advertising claims made on branded E-Cigarette reported that it is marketed as a healthier and cheaper alternative to conventional smoking. And the appeal of the wide array of flavors, perception of modernity, enhanced social appearance and celebrity use, and avoidance of smoking policy were appealing to many [9].

Based on largely observational data on the current outbreak, the majority of reported cases were young males [10–13]. The commonly reported clinical symptoms include fever, chills, and a headache. Respiratory symptoms include shortness of breath, cough, and chest pain. Gastrointestinal symptoms include nausea, vomiting, diarrhea, and abdominal pain [10–13]. Fever was present in 29%, tachycardia in 64%, and tachypnea in 43%, with oxygen saturation < 88% in 31% of the cases in one report [10]. There are no clear guidelines regarding the management of EVALI, although glucocorticoids and empiric systemic antimicrobial therapy have been prescribed in the majority of cases [10, 11, 13]. The improvement in respiratory symptoms has been attributed to glucocorticoids [10, 12].

86% of the 867 EVALI patients report using THC-containing products in the three months preceding symptom onset with mild elevations in white cell count, sedimentation rate, and procalcitonin [14]. Both of our patients were young males who vaped THC and met a “confirmed” diagnosis of EVALI set by the CDC. The clinical characteristics and presentation were consistent with previously reported cases [15].

A wide range of imaging patterns has been described in EVALI [16, 17], including hypersensitivity pneumonitis, diffuse alveolar damage, acute lung injury, acute eosinophilic pneumonia, organizing pneumonia, lipoid pneumonia, and giant cell interstitial pneumonia. Imaging obtained during the acute phase for both our patients demonstrated diffuse ground-glass opacities slightly more prominent at the lower lung zones, with subpleural and peribronchovascular sparing. Our patients had a pattern most consistent with organizing pneumonia, which has been described in the EVALI literature. Peribronchovascular sparing, as was seen in our patients, is not a well-described feature that may represent clearing by the peribronchovascular lymphatics related to a subacute process with vaping. Of note, 100% of the cases in the report of 53 cases had bilateral infiltrates on chest radiograph or CT [10].

The pathology of EVALI continues to be poorly understood with no specific histopathological findings. A recent review described lung biopsies from 17 patients with either a “confirmed” or “probable” diagnosis of EVALI. All showed a pattern of acute lung injury, including acute fibrinous pneumonitis, diffuse alveolar damage, or organizing pneumonia. While other reports suggested an exogenous lipoid pneumonia-like process, the authors suggested that the patterns resemble a chemical pneumonitis [18]. Nevertheless, both findings do not provide clarity while making a histological diagnosis for EVALI. Our second patient showed similar histopathology, as described in the literature, with findings suggestive of acute pneumonia/inflammation with macrophages containing blackish brown pigmented material. Our first patient did not show any histological pattern of lung injury despite meeting a “confirmed” diagnosis of EVALI by the CDC [15]. However, the biopsy showed predominantly bronchial mucosa and not adequate alveolar tissues; thus, whether the patient had normal lung histopathology is inconclusive.

E-Cigarette aerosol contains different harmful substances, including carbonyls, volatile organic compounds, toluene, benzene, and heavy metals [19, 20]. Their constituents undergo thermal decomposition by the metallic coils, which then release new compounds that can be toxic [21]. In one study, E-Cigarette products contained microbial toxins, which included endotoxin concentrations above the limit of detection (LOD) in 17 of the 75 products tested and glucan concentrations greater than the LOD in 61 of 75 products tested [22]. Certain pesticides, such as chlorpyrifos ethyl and trifluralin, have been detected in high concentrations in some E-Liquid samples [23].

Triantafyllou et al. described several proposed mechanisms of EVALI, which include alteration in the expression of bronchial epithelial proteins, inducing airway remodeling, and macrophage activation [12]. Marijuana smoke inhalation has been associated with damage to the pulmonary epithelial barrier, and even the flavoring component has been linked to lung injury [23]. Compounds such as diacetyl, 2,3-pentanedione, and acetoin can impair ciliary function and have been evaluated and linked to bronchiolitis obliterans, the popular term being “popcorn lung,” which causes severe respiratory diseases among workers inhaling these heated vapors [24–26].

Recently, samples of THC-containing products have been analyzed to identify the potential contributing substance resulting in harmful effects on the lung. Different substances were identified, which have included vitamin E acetate, medium-chain triglyceride oil (MCT oil), and other lipids [27] (Personal communication, D.T. Heitkemper, FDA Forensic Chemistry Center, November 2019). Vitamin E acetate, in particular, has been identified in the majority of the THC cartridge samples tested in various states and facilities [14, 28]. Vitamin E acetate is a compound that is used as an additive to E-Cigarette and as a thickening agent in THC products [29]. Vitamin E acetate was further implicated when a breakthrough report from CDC on November 8, 2019, showed that it was detected in 100% of the BAL samples of the 29 cases with EVALI that were sent to the CDC from 10 different states across the United States, while THC was identified in 82% and nicotine in 62% of the samples. Of note, no other suspected substances (plant oils, MCT oil, petroleum distillates, and diluent terpenes) were detected in those 29 BAL samples [30]. Inhaling vitamin E is not as benign as ingesting vitamin E, as it can impair lung function [31]. This provides strong evidence that vitamin E acetate might be a potential agent, though further studies are needed to validate this link.

## 4. Conclusion

EVALI can be devastating and even fatal, as was seen by the recent outbreak. Although various substances have been investigated, vitamin E acetate appears to be a risk factor. More data is needed to establish causality. With this report of two cases with radiologic and pathological findings, we hope to enrich the growing database and increase our knowledge of this disorder.

## Figures and Tables

**Table 1:** Lab work on admission.

	Case 1	Case 2
*Infectious workup*		
White cell count	11.3K/*μ*l	14.3K/*μ*l
Sedimentation rate (ESR)	80 mm/hr	91 mm/hr
Procalcitonin	0.21 ng/ml	0.40 ng/ml
Legionella urine antigen	Negative	Negative
Pneumococcal urine antigen	Negative	Negative
Histoplasma urine antigen	Negative	Negative
Blood culture for aerobic & anaerobic growth	Negative	Negative
Respiratory viral pathogen screen	Negative	Negative
Sputum culture	Negative	Negative
Sputum AFB smear	Negative	Negative
HIV AG/AB	Negative	Negative
ANCA	Negative	Negative
1,3-b-d-Glucan	Negative	Negative
BAL cell count and differentials	Normal	Normal
BAL total nucleated cells	440/*μ*l	640/*μ*l
BAL RBCs	<10.000/*μ*l	<10.000/*μ*l
BAL neutrophils	49%	39%
BAL lymphocytes	28%	5%
BAL monocytes	17%	8%
BAL phagocytes	6%	48%
BAL fungal culture with special	Negative	Negative
BAL vulture viral reflex	Negative	Negative
BAL bacterial culture	Negative	Negative
BAL Aspergillus antigen	Negative	Negative
*Miscellaneous workup*		
Lyme disease serology	Negative	
Rapid strep A	Negative	
West Nile antibodies	Negative	
Glomerular basement membrane (GBM) antibodies	Negative	
Mononucleosis screen	Negative	
Complement levels	Normal	
LDH level	Normal	
ProBNP	74 pg/ml	
CRP	>375 mg/l	
Coccidioides antibodies		Negative
Hypersensitivity pneumonitis panel		Negative
BAL AFP culture and smear	Negative	
BAL PCR for Legionella	Negative	

## Abbreviations

EVALI: E-Cigarette or vaping product use-associated lung injury
THC: Tetrahydrocannabinol
CBD: Cannabidiol
CDC: Centers for Disease Control and Prevention
CXR: Chest X-ray
CT: Computed tomography
BAL: Bronchoalveolar lavage
HRCT: High-resolution computed tomography
ESR: Sedimentation rate
ANCA: Antineutrophil cytoplasmic antibody
AFB: Acid-fast bacillus
HIV: Human immunodeficiency virus
AG: Antigen
AB: Antibody
GBM: Glomerular basement membrane
LDH: Lactate dehydrogenase
BNP: B-Natriuretic peptide
PCR: Polymerase chain reaction
LOD: Limit of detection
MCT: Medium-chain triglyceride.
